# 

**Published:** 1902-09

**Authors:** 


*The following donations have been received since the publication of the List in June:*

*August 31st, 1902.*

The Executors of the late Joshua Smith Beddome (1) ... ... ... ... ... ... ... ... ... ... 8 volumes.

W. Fairbanks, M.B. (2) ... ... ... ... ... ... 28 „

Mrs. E. Long Fox (3) ... ... ... ... ... ... 19 „

L. M. Griffiths (4) ... ... ... ... ... ... ... 3 „

A framed portrait has been presented by Dr. D. S. Davies, and unbound periodicals have been received from Mrs. Long Fox.

### FORTY-FIFTH LIST OF BOOKS.

The titles of books mentioned in previous lists are not repeated.

The figures in brackets refer to the figures after the names of the donors, and show by whom the volumes were presented. The books to which no such figures are attached have either been bought from the Library Fund or received through the *Journal*.

**Abercrombie, J.**... *On the Intellectual Powers* ... ... ... (1) 6th Ed. 1836
„ *The Culture and Discipline of the Mind*... (1) 3rd Ed. 1837

**Astruc, [J.]** ... *Treatise on the Venereal Disease* (Tr. by S. Chapman) (2) 2nd Ed. 1770

**Ballantyne, J. W.** *Manual of Antenatal Pathology and Hygiene* ... ... 1902

**Ballard, T.** ... *Diseases Peculiar to Infants and Mothers* ... ... (2) 1860

**Baly and W. W. Gull, W.** *Reports on Epidemic Cholera* ... ... ... (2) 1854

**Barwell, R.** ... *Diseases of the Joints* ... ... ... ... ... ... ... (4) 1861

**Bastian, H. C.** ... *Studies in Heterogenesis,* Part II. ... ... ... ... ... ... 1901

*Bath, Medical Guide to the Hot Mineral Baths of* ... ... ... ... ... ... ... 1901

**Baudelocque, [J. L.]** *L'Art des Accouchemens,* Tomes i., ii. ... ... (2) 1781

**Benedikt, M.** ... *D'Anthropométrie Cranio - Céphalique* (Tr. par P. Keraval) ... ... ... ... ... ... ... ... ... (3) 1889

**Bigg, H.** ... ... *Caries of the Spine* ... ... ... ... ... ... ... ... ... 1902

**Billard, C. M.** ... *Maladies des Enfants* ... ... ... ... (2) 3e Éd. 1837

**Birkett, J.** ... ... *The Diseases of the Breast* ... ... ... ... ... (2) 1850

**Blundell, J.** ... *Diseases of Women.* (Ed. by T. Castle) ... ... (2) 1837

**Buchan, W.** ... *Domestic Medicine* ... ... ... ... ... ... (1) 11th Ed. 1790

**Burns, J.** ... ... *The Principles of Midwifery* ... ... ... (2) 9th Ed. 1837

**Canney, L.** ... ... *Typhoid the Destroyer of Armies and its Abolition* (3) 1901

**Cooper, S.** ... ... *First Lines of Surgery* ... ... ... (2) [2nd] Ed. 1809

**Cotton, R. P.** ... *Phthisis and the Stethoscope* ... ... ... ... ... (2) 1851

**Cunningham, D. J.** [Ed.] *Text-Book of Anatomy* ... ... ... ... ... 1902

**Day, H.** ... ... *Clinical Histories with Comments* ... ... ... ... (3) 1866

**Dorland, W. A. N.** *The American Illustrated Medical Dictionary* ... ... 1900

**Drake, N.** ... ... *Mornings in Spring* ... ... ... .. ... (1) 2 vols. 1828

**Duchenne, G. B.** *Album de Photographies pathologiques* ... ... ... (3) 1862

**Fyffe, W. J.** ... *An Address on the Preservation of Health During School Boy Life* ... ... ... ... ... ... ... ... ... (3) [N.D.]

**Good, J. M.** ... *Song of Songs* ... ... ... ... ... ... ... ... ... (1) 1803

,, ... *The Book of Job* ... ... ... ... ... ... ... ... (1) 1812

**Gould and J. C. Warren, A. P.** [Eds.] *International Text-Book of Surgery.* Vol. I. 1900

**Gull, W. Baly and W. W.** *Reports on Epidemic Cholera* ... ... ... (2) 1854

**Harrison, R.** ... *Some Points in Practical Surgery Suggested by the Study of the Life and Work of John Hunter* ... ... ... 1902

**Heath, C.** ... ... *Minor Surgery and Bandaging* ... ... (2) 3rd Ed. 1866

**Hills, M.** ... ... *A Treatise on the Operation of Cupping*... ... ... (3) 1839

**Hoffman, F.** ... *Opera Omnia Physico-Medica.* Tome v.; Suppl. ii., Parts i., ii., iii. (2) 1748–53

**Hood, P.** ... ... *Diseases of Children* ... ... ... ... ... ... ... (4) 1845

**Knapp, P. C.** ... *Intra-Cranial Growths* ... ... ... ... ... ... (3) 1891

**Laurie, J.** ... ... *Homœopathic Domestic Medicine* ... .. ... ... (2) 1851

**Lendon, A. A.** ... *Clinical Lectures on Hydatid Disease of the Lungs* .. 1902

**Leyden, E.** ... *Klinik der Rückenmarks-Krankheiten,* Bd. 1... ... (3) 1874

**Mackenzie, F. W.** *Phlegmasia Dolens* ... ... ... ... ... ... ... (3) 1862

**Marsh, H.** ... ... *Clinical Essays and Lectures* ... ... ... ... ... ... 1902

**Mead, R.** ... ... *Medical Works* ... ... ... ... ... ... ... ... (2) 1762

**Moore, G.** ... ... *Health, Disease and Remedy* ... ... ... ... ... (2) 1850

**Ogle, J. W.** ... *Miscellaneous Contributions to the Study of Pathology* (3) 1868

**Piper, H.** ... ... *Scriftproben von Schwachsinnigen resp. idiotischen Kindern* ... ... ... ... ... ... ... ... ... (3) 1893

| | | | |
|---|---|---|---|
| **Pulte, J. H.** | *Homœopathic Domestic Physician* | (2) 3rd Ed. | 1855 |
| **Quincy, J.** | *Lexicon Physico-Medicum* | (2) 11th Ed. | 1794 |
| **Remak, E.** | *Uber die Localisation atrophischer Spinallähmungen* | (3) | 1879 |
| **Riverius, L.** | *The Universal Body of Physick* (Tr. by W. Carr) | (2) | 1657 |
| **Rowe, G. R.** | *Disorders of Females and Children* | (2) | 1857 |
| **Shaw, W. N.** | *Ventilation as a Dynamical Problem* | | 1902 |
| **Sibson, F.** | *On the Causes which Excite and Influence Respiration in Health and Disease* | (3) | [N.D.] |
| **Stilling, J.** | *Pseudo-isochromatische Tafeln für die Prüfung des Farbensinnes* | (3) | 1883 |
| **Smith, W. T.** | *Leucorrhœa* | (2) | 1855 |
| **Sydenham, T.** | *The Whole Works of* (Tr. by J. Pechey) | (2) | 1722 |
| **Thompson, C. J. S.** | *Aids to Practical Dispensing* | 3rd Ed. | 1902 |
| **Warren and A. P. Gould, J. C. [Eds.]** | *International Text-Book of Surgery.* | Vol. I. | 1900 |
| **West, S.** | *Diseases of the Organs of Respiration* | 2 vols. | 1902 |
| **Williams. J.** | *Insanity* | (2) 2nd Ed. | 1852 |
| **Wilson, J.** | *The Water Cure* | (2) 3rd Ed. | 1843 |
| **Wood, H. C.** | *Fever* | (3) | [N.D.] |

### TRANSACTIONS, REPORTS, JOURNALS, &c.

| | | |
|---|---|---|
| American Medicine | Vol. II. | [1901] |
| American Pediatric Society, Transactions of the | Vol. XIII. | 1901 |
| Berliner klinische Wochenschrift | (3) | 1873 |
| Bristol Medico-Chirurgical Journal | Vol. XIX. | 1901 |
| Bristol Port Sanitary District—Annual Report for 1901 | | 1902 |
| Catalogue of Books, The English | (4) | 1857–61 |
| Dictionary of Bathing Places, Bradshaw's | | 1902 |
| Edinburgh Medical Journal, The | N.S., Vol. XI. | 1902 |
| Hospital, The | Vol. XXXI. | 1902 |
| HOSPITALS— | | |
| Johns Hopkins Hospital Reports | Vol. X., Nos. 3-4-5 | 1902 |
| Middlesex Hospital Reports for 1900 | | 1902 |
| St. Thomas's Hospital Reports | N.S., Vol. XXIX. | 1902 |
| Library, The | Vol. II. | 1901 |
| Library Association Year Book, The | | 1902 |
| Lunacy, Report of the Metropolitan Commissioners in | (2) | 1844 |
| Medical Chronicle, The | 4th Ser., Vol. I. | [1901] |
| Rhode Island Medical Society, Transactions of the | Vol. VI., Part III. | 1902 |

---

In the paper entitled "Some Things of General Interest in the Bristol Medical Library,"[1] which Mr. L. M. Griffiths read before the Library Association, some bibliographical points are of medical interest.

The Bristol Library Society, dating from 1772, in course of time had practically taken possession of the premises of the City Library in King Street. In 1855, when it was known as the Bristol Library, it

[1] *Library Association Record*, June and July, 1901.

moved to premises near the top of Park Street, which are now being demolished to make room for the Art Gallery, and in 1856 it received the collection of books which had been formed by the doctors under the title of the Bristol Medical Library, which had been established in Orchard Street. The transfer was made on the condition that its efficiency should be constantly maintained. In 1867 the Society allied itself to the Bristol Philosophical and Literary Institution which had been founded in 1817, and in 1871, in the building known as the Museum and Library, the work of the two societies was carried on conjointly. Many difficulties beset the new institution; the stipulation about the medical books was not kept, and in 1893 Sir Charles Wathen, then Mayor of Bristol, undertook to pay the debts of the institution on condition that the shareholders presented the building and its contents to the city. The present Medical Library in Bristol, begun in 1891, now has the custody of the books which, belonging to the former Medical Library, have passed through so many vicissitudes of ownership.

The Bristol Medical Library does not possess a Caxton or a book printed by any of his contemporaries, or even a De Worde or a Pynson, but it has a book printed in the year IX.

In 1834 James Atkinson, a surgeon of York, issued a Medical Bibliography, including only A and B, which he did not intend to carry further. He says: "Bibliography is a dry occupation—a *caput mortuum*—it is a borrowed production, which brings very little grist to the mill; and so difficult and tedious is the object, of laying before our eyes all the real or reported copies or editions of the works enumerated, that almost every line of our reports may be suspected of falsehood. How are we to collect, how to produce, how to examine, the originals? Many books are so scarce, so sequestered in private hands, or in the mansions of the great, that even the keen eyes of luciferous booksellers cannot find them. And if they cannot, who the deuce can?" To render bibliography less dry, he added to his lists of books and periodical literature, which are very commendable, observations on the authors. The style of these may be gathered from two instances. Of Dr. Beddoes he says: "As an author, he appears to have been always in a hurry to reach the mart of novelty and invention, lest others should arrive there before him; so that it became, through life, a perpetual tilt and tournament for fame." He considers that Benjamin Brodie, who was more than a distinguished surgeon, has been fortunate in regard to his surgical pursuits, and adds that "generally speaking, the surgeon who exerts himself, however justly, as a philosopher and a naturalist, is often superseded in the surgical department by surgeons his inferiors. The idle world seldom gives a man credit for excellence in two attainments. Ex. gr. I plays a bit at top o' the fiddle; so neighbours say I can do nothing else." About bibliography generally Atkinson says: "No man's industry is mis-spent, if he merely clear the obstruction from any path; and the very attempt to show what is right, frequently exposes that which is wrong; so that the immediate blunders of one person rectify those of another: and he ever must deserve well of society who attempts improvement."

Remembering the difficulties and dangers of subject-classification, and our own mistakes, we shall be lenient to the errors of others, and "forbear to judge; for we are sinners all;" but it is difficult to forgive the British Museum for placing Ziemssen's *Cyclopædia of Medicine* in a list of bibliographical works, or for putting Holden's *Landmarks*, a book which deals with surface anatomy, under the heading of "History of Medicine," or worst of all, for classing under "Obstetrics," a work on

"Child Labour," especially as it adds immediately after the entry that it was issued by the American Economic Association.

There is no better instance of careful bibliography than the *Index-Catalogue of the Library of the Surgeon-General of the United States Army.* Sixteen volumes were issued in the first series, and the sixth volume of the second series is in the letter H. The references to Cholera in the two volumes in the two series may be taken as an example of its value. In the first of these the titles cover 152 pages, and in the second 102, a fairly good start for the man who wants to get up the subject, It is only right to mention the *Index Medicus*, a monthly classified record of current medical literature, a work which grew out of the *Index-Catalogue.* After twenty-one years of existence, during which it passed through many difficulties, it came through insufficient support to an end. It is a book simply indispensable to the librarian and the student. The most expert cataloguer must admit its thoroughness and its splendid accuracy. Its place has been excellently filled by *Bibliographia Medica*, working on almost exactly similar lines, but incorporating the Dewey system. It is issued by the *Institut de Bibliographie* of Paris, and fortunate is the branch of literature that has such an invaluable help.

A puzzling bit of bibliography comes before us in reference to the anatomical work of Bidloo, who in 1685 issued from Amsterdam his *Anatomia Humani Corporis.* In 1698 the same plates with reference in red ink and nine additional plates were printed at Oxford, and on the engraved title the portion indicating Bidloo's authorship is covered by a slip declaring it to be "The Anatomy of Humane Bodies by Willm. Cowper, Surgeon," and this is so neatly pasted over as to escape recognition until very closely examined. Cowper, in an address to the reader, says: "These Figures were Drawn after the Life, by the Masterly Painter G. de Lairess, and Engrav'd by no less a Hand, and Represent the Parts of Humane Bodies far beyond any Exstant; and were some time since Publish'd by Dr. Bidloo, now Professor of Anatomy in the University of Leyden," and the theory is that Cowper bought the plates and considered that the additions he made were sufficient to justify him in calling the work his own. But this hardly seems to be ideal bibliographical morality, and the view which Bidloo took of the transaction may be gathered from a fifty-four page pamphlet which he issued from Leyden in 1700 entitled *Gulielmus Cowper criminis literarii citatus, coram tribunali Nob. Amp. Soc. Brit.-Reg.* Cowper appears to have made a tardy recognition of Bidloo in his Εὐχαριστία, *in qua dotes plurimæ et singulares Godefridi Bidloo, perita anatomica*, [etc.]. issued in 1701. But the mystery becomes more involved when we look at a book containing the same plates which was issued at Leyden in 1737 under the supervision of C. B. Albinus who described the work as the second edition of Cowper's book. This seems extraordinary when we remember that Albinus was Professor of Medicine, Anatomy, Surgery and Practice in the University of Utrecht, and could hardly have been ignorant of the incidents connected with Bidloo and Cowper.

### Local Medical Notes.

**University College, Bristol.**—The following students of the College have been successful in recent examinations, viz.:—Mr. D. T. Price in the Final, and Mr. T. Pratt in the Intermediate, M.B. Examinations of the London University; Mr.

C. Corfield in the M.R.C.S. and L.R.C.P.; and Messrs. C. J. Taylor and W. P. Taylor in the L.S.A. Examinations.

D. T. Price, M.B. Lond., has been appointed Junior House Surgeon to the Salop Infirmary, Shrewsbury; J. C. Clayton, M.R.C.S., L.R.C.P., Junior House Physician, Wolverhampton and Staffordshire General Hospital.

**The Bristol General Hospital.**—Now that the work is completed, and the various artificers who have for so many months graced the out-patient department of the Hospital with their presence and with their appurtenances—their scaffoldings, implements of labour, bricks, mortar, and other raw material—have at last departed, the great improvements which have been effected in the department can be satisfactorily estimated and appreciated. Those who remember the old arrangements for the reception of casualties, the narrow and shabby entrance, the dull and awkward passages, the small, inconvenient, and often crowded casualty room, will admit that those arrangements have been vastly improved. Through wide and folding-doors the out-patients now pass into a spacious and imposing hall of entrance, bright and cheerful in aspect. Turning to the left beyond this they pass to two large, comfortable and warm waiting-rooms, which communicate with the casualty-rooms. The new main casualty-room has been constructed on modern and aseptic principles, and the excellence of the arrangements leaves little to be desired. Opening into the main room is a second one of good size, available for special cases or for operations.

One of the most striking of the new additions is the chamber set apart for electrical and hydropathic treatment. In it are found numerous baths of various forms, each supplied elaborately with ingress and egress water-pipes and stop-cocks, and with refined electrical adjuncts. Here also are installed some of the most modern Finsen light lamps. But it would really require the pen of a specialist to adequately describe the charms of this attractive room. Two other rooms have, in addition, been set apart for radiography.

The old dispensary and adjacent waiting-rooms have been replaced by new structures of more convenient, comfortable and spacious design. The special rooms of the ophthalmic and laryngological department have shared in the improvements proportionately to the departments already mentioned, and the main room, the dark room, and the adjacent waiting-rooms are all excellent. All the new rooms are well supplied with the electric light, which light, indeed, is now used throughout the Hospital.

We congratulate the committee upon having entered upon and completed a far-reaching scheme of improvement. We trust a similar discretion and zeal will shortly lead them to take in hand the renovation on modern principles of the existing operating theatre and the construction of a second theatre; the want of the latter is becoming more and more felt.

**The General Hospital Fête.**—This *fête* was held at the Clifton Zoological Gardens on June 12th and following days. The fourth day (Monday) was an extra day, arranged in the hope of compensating in some degree for the losses sustained through the bad weather of the other days. If the Committee of Management had made special efforts to secure three of the worst possible days in the season for the *fête*, they could hardly have selected days more suitable for the purpose than the fateful days which they actually did select. There was an almost continuous downpour of rain from the time of the opening ceremony until the evening of the last day. Most things animate and inanimate became soaked, and the mud underfoot, and indeed that on foot and on skirt, was a thing never to be forgotten. It became finally almost dangerous to embark upon the sea of mud to which the formerly well-kemped lawns had given place. On the evening of the last day the weather improved, and, encouraged by it, a large number of people visited the grounds that evening, showing what a great success was likely to have been achieved under favourable conditions. The admirable spirit, however, of those who took an active part in organising and conducting the *fête* was such that a great success was made of it in spite of the incorrigible misbehaviour of the weather.

The amount taken in the Gardens was about £800. The treasurers had previously collected subscriptions amounting to £1,000. Mr. Spark Evans, with great generosity, recently gave £500 to the funds, which is some compensation for the loss due to the bad weather. After all expenses have been deducted, therefore, the Hospital will gain some £2,000 as a result of the labours of those who devoted so much time and trouble to the organisation of the *fête* and to enlisting the sympathy and support of generous subscribers.

